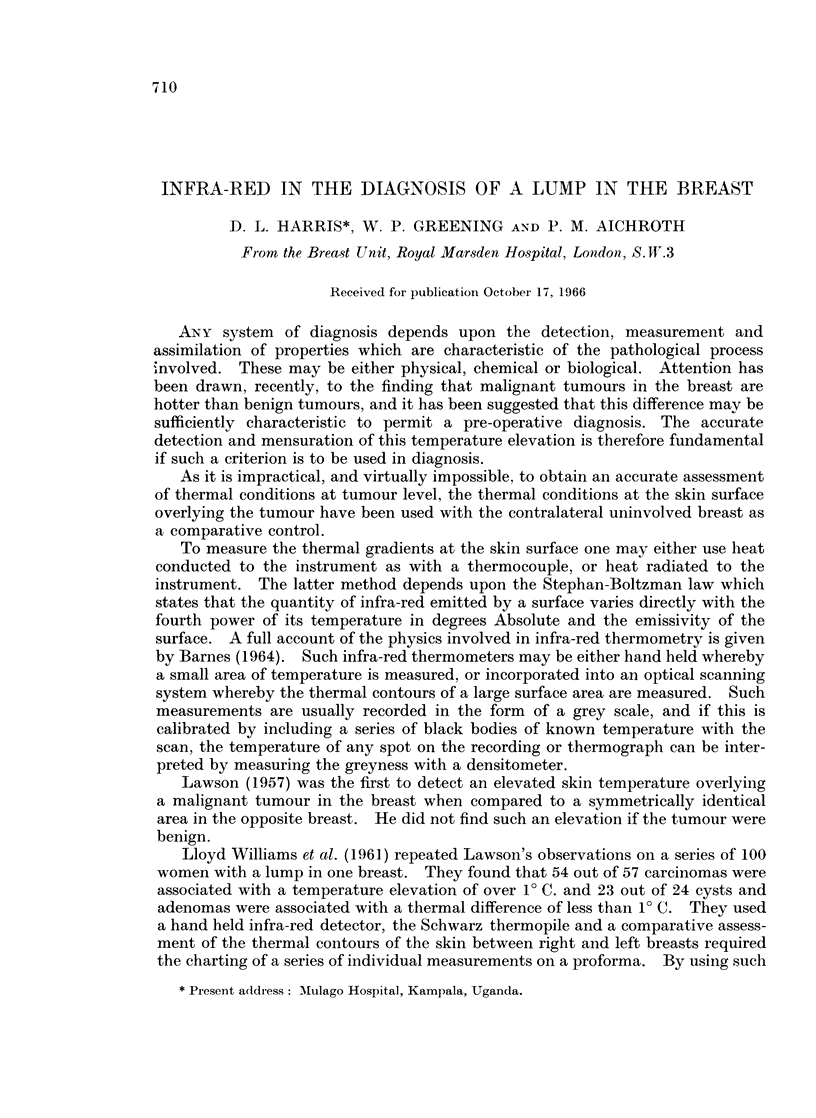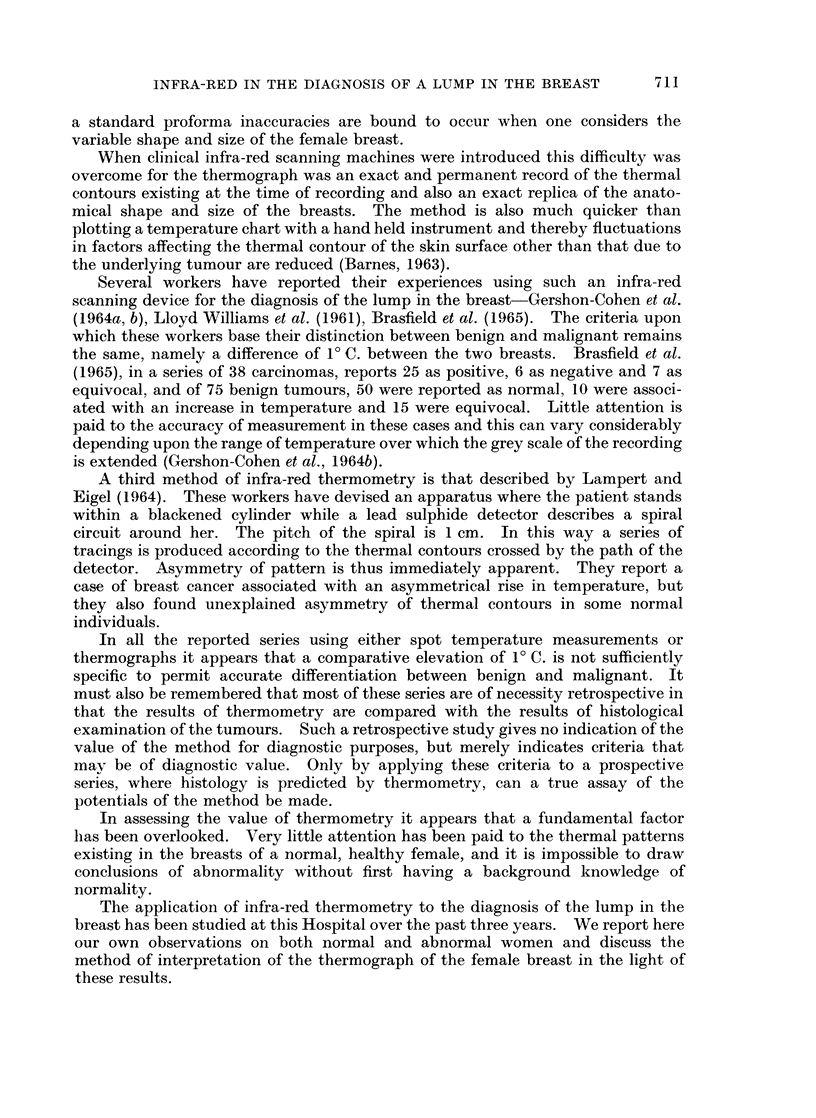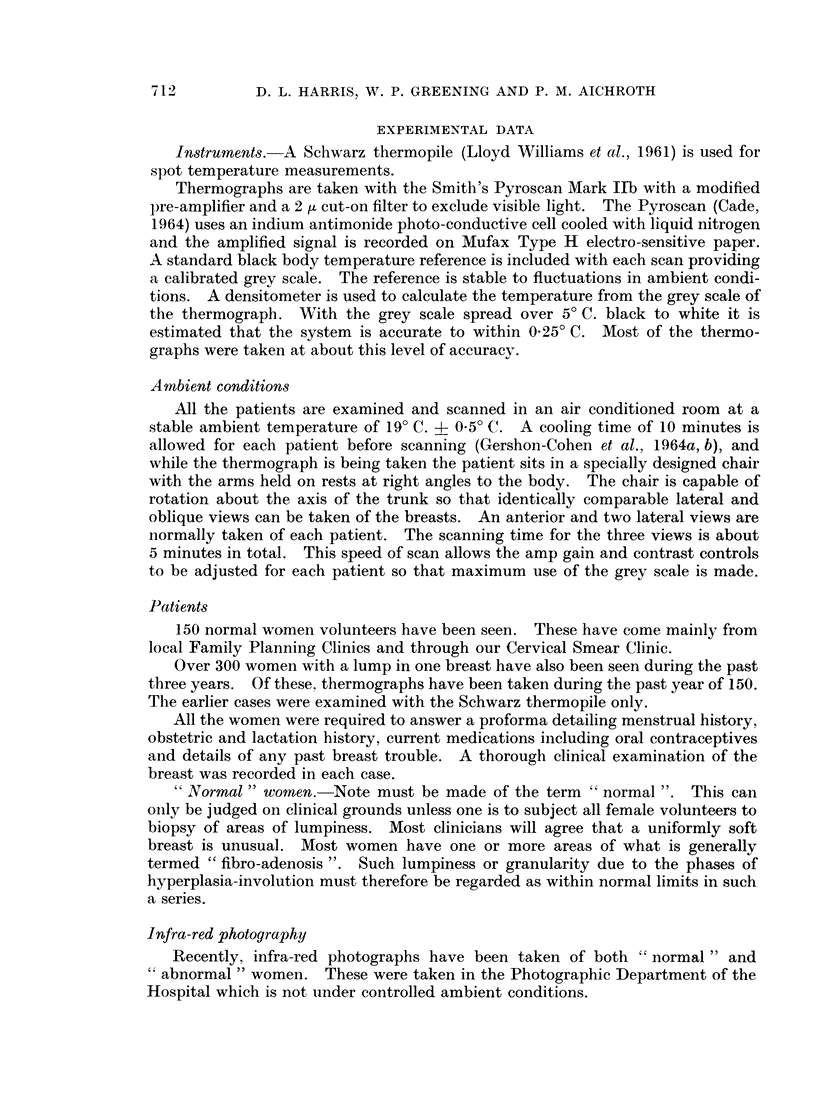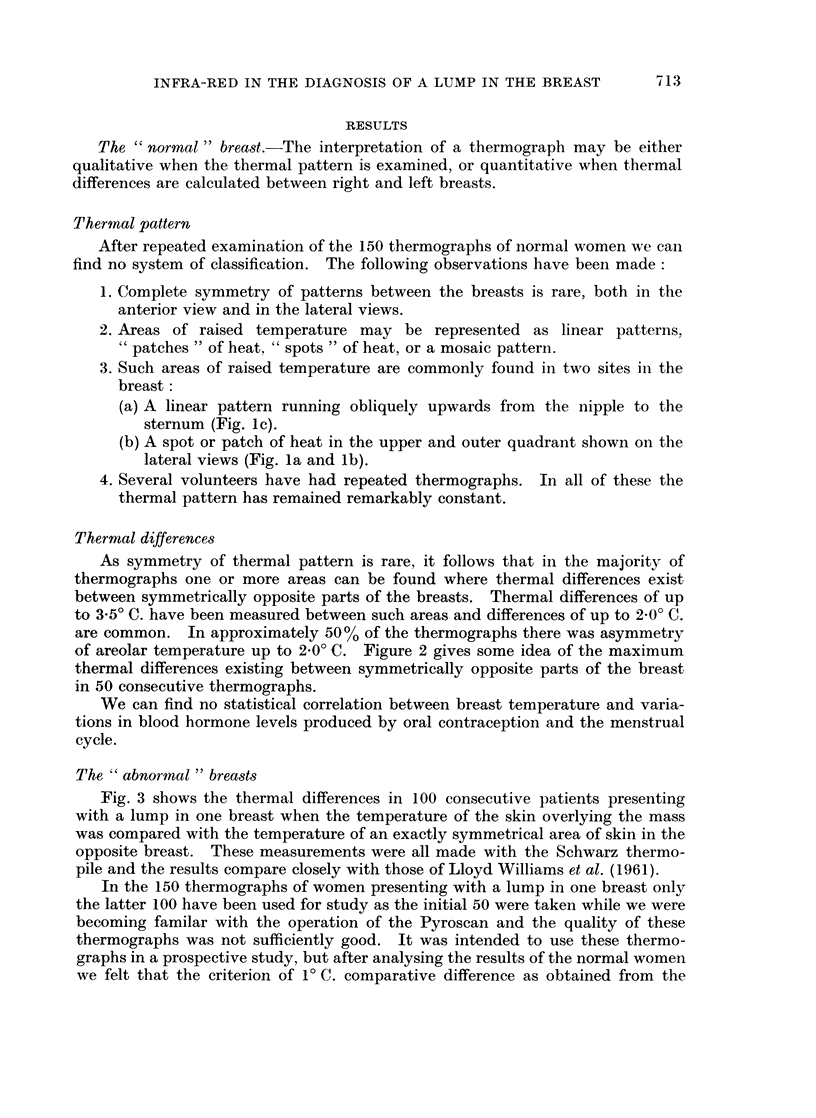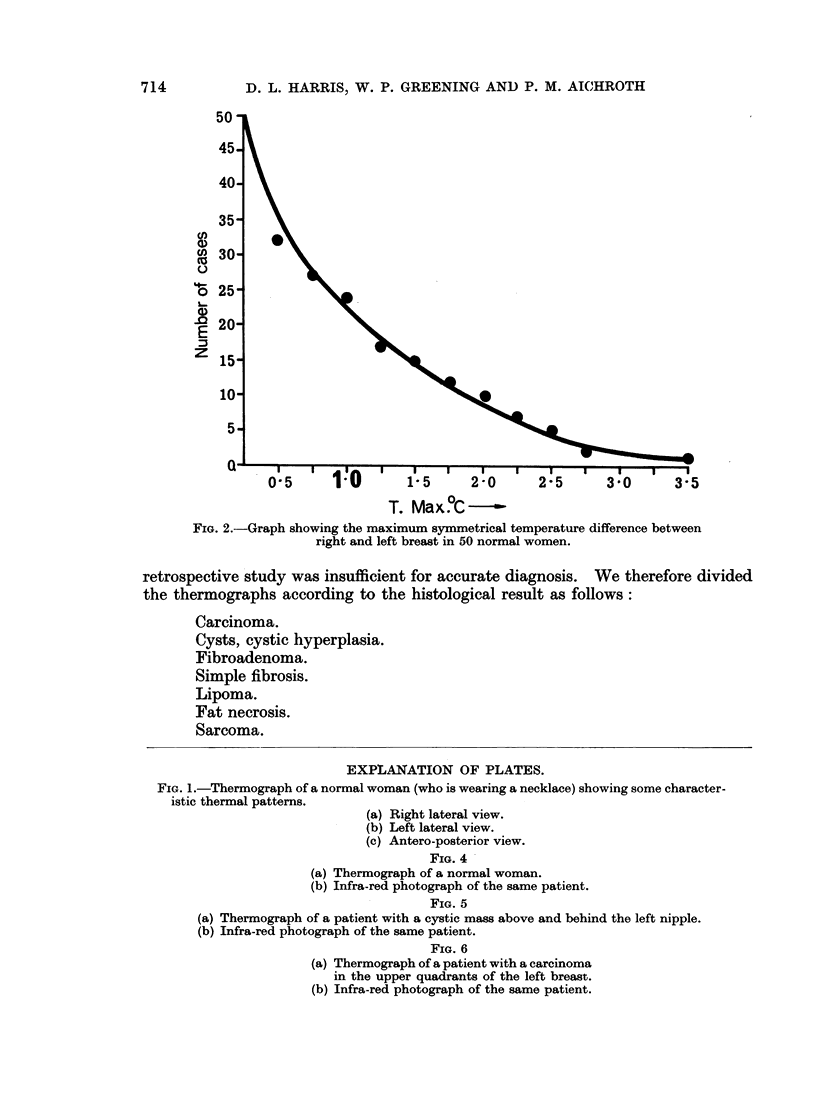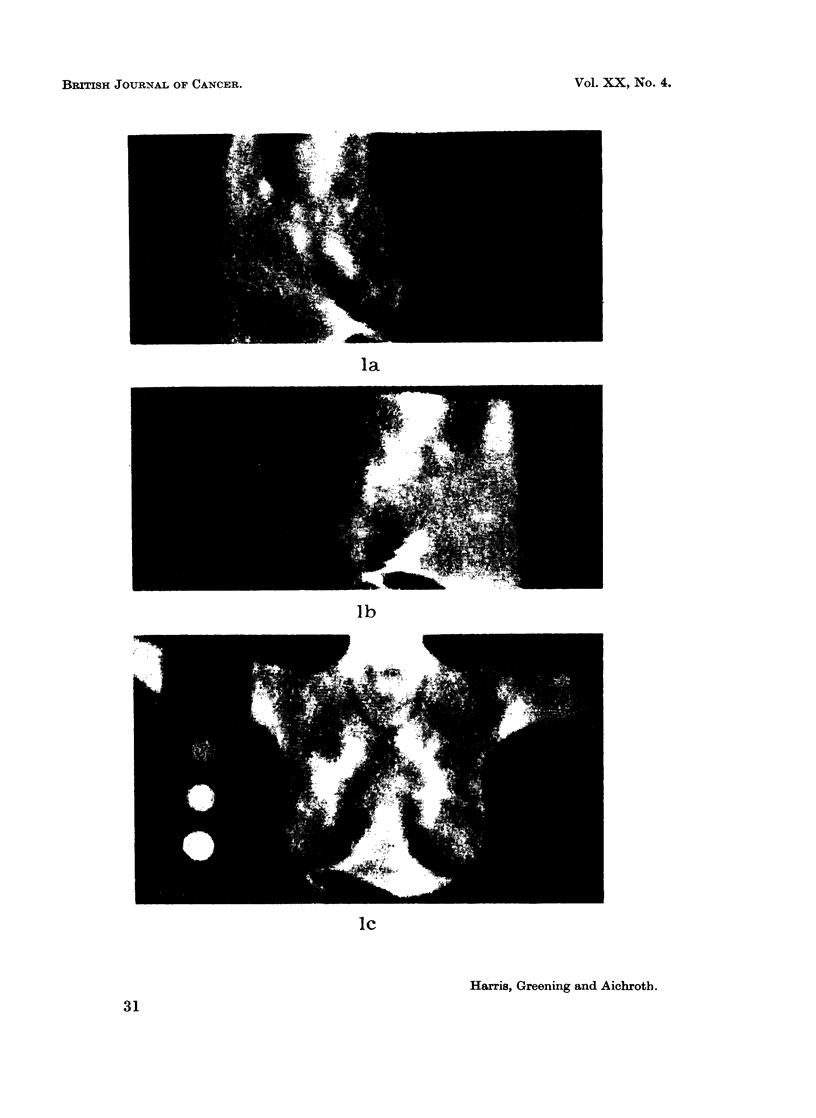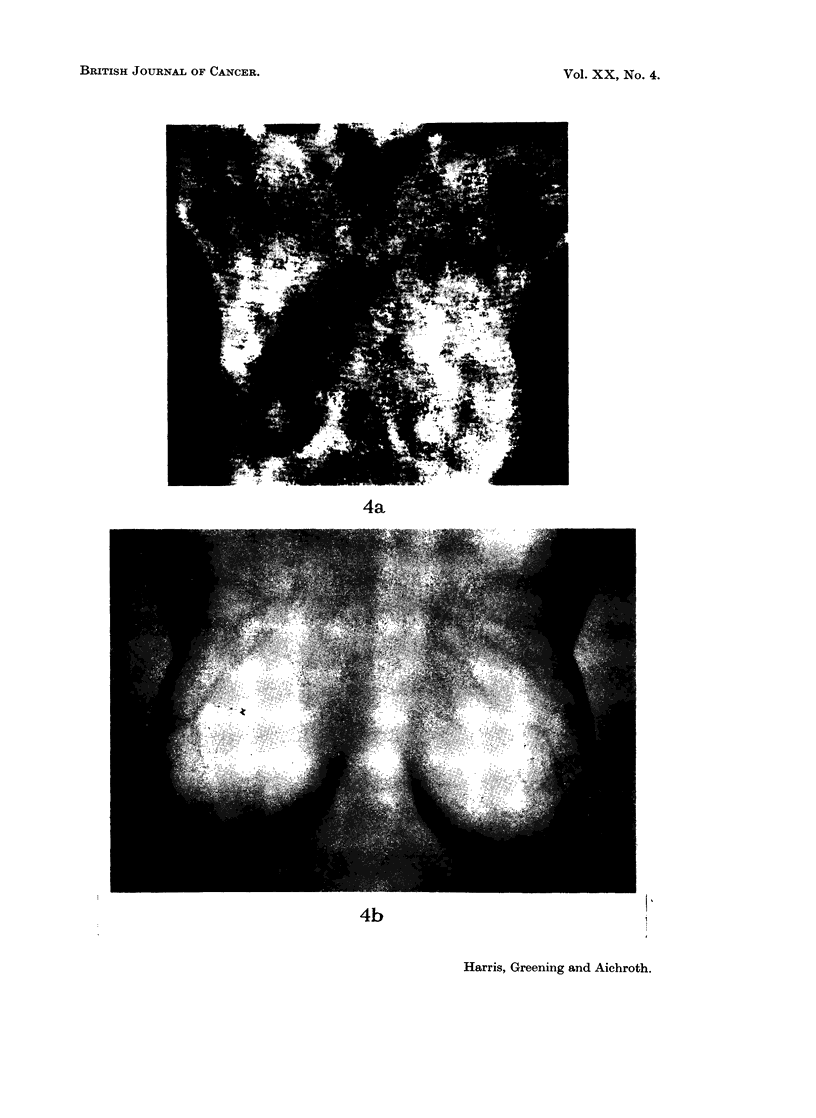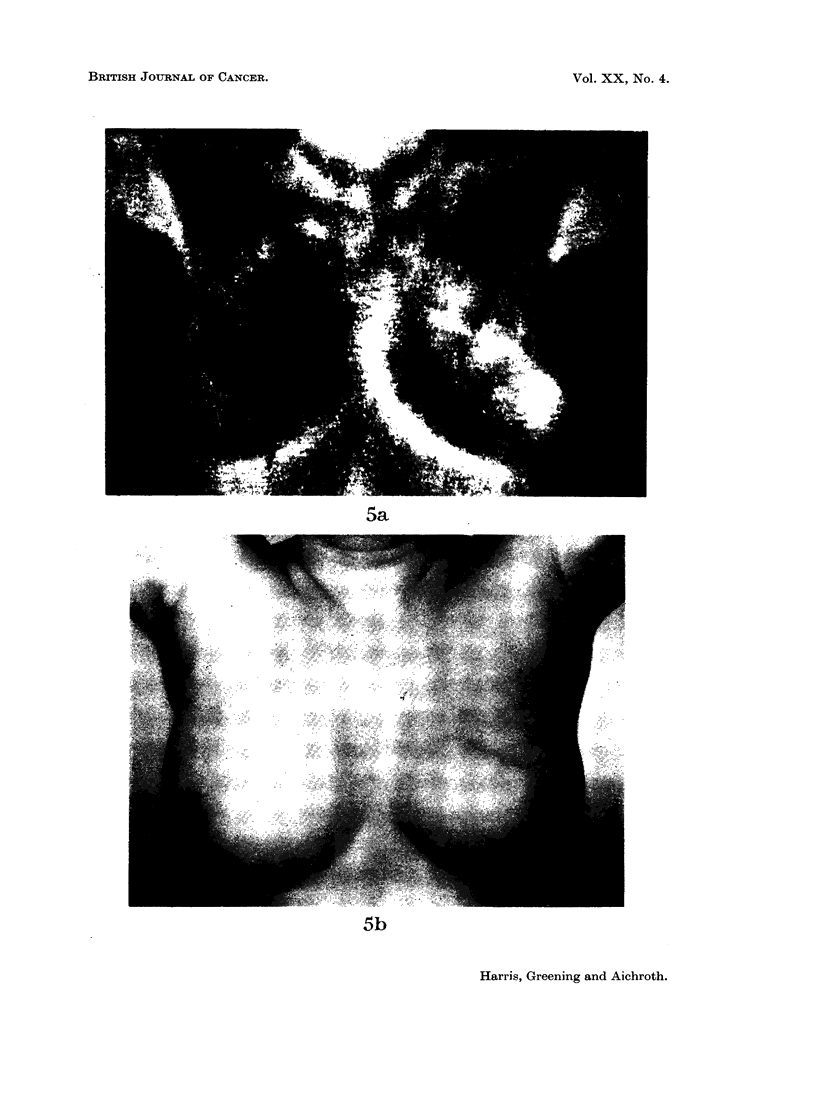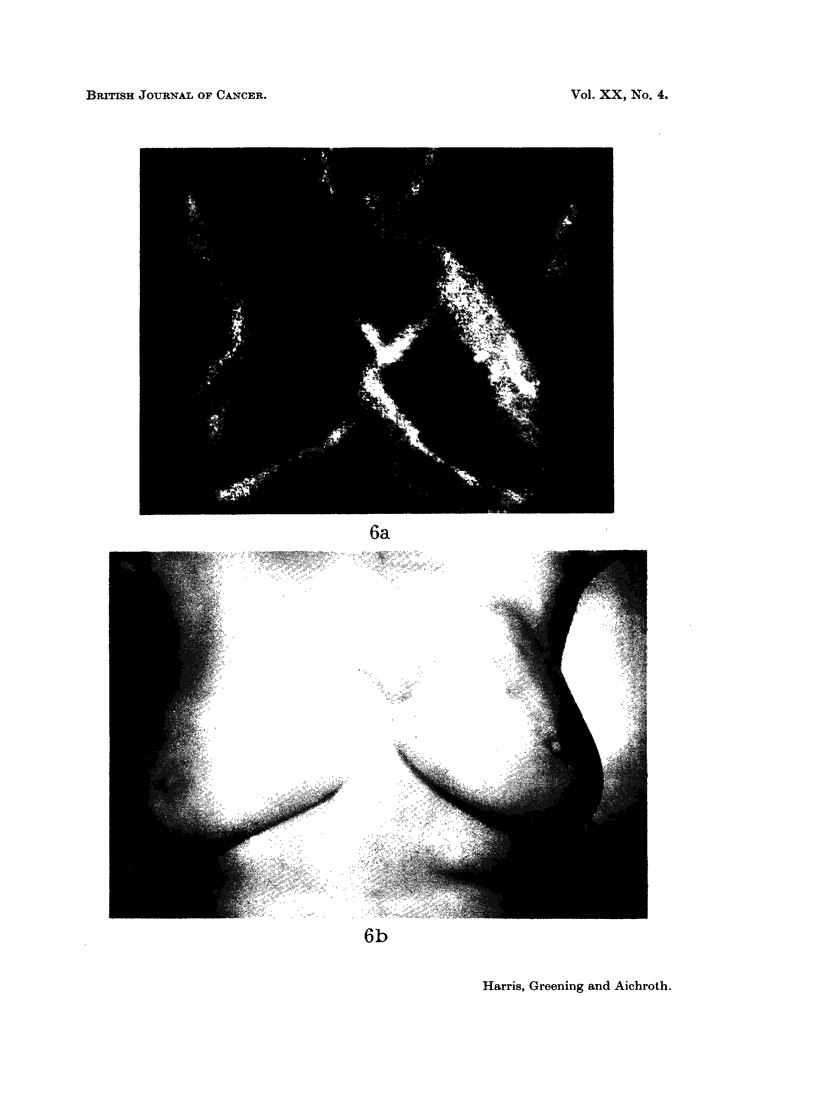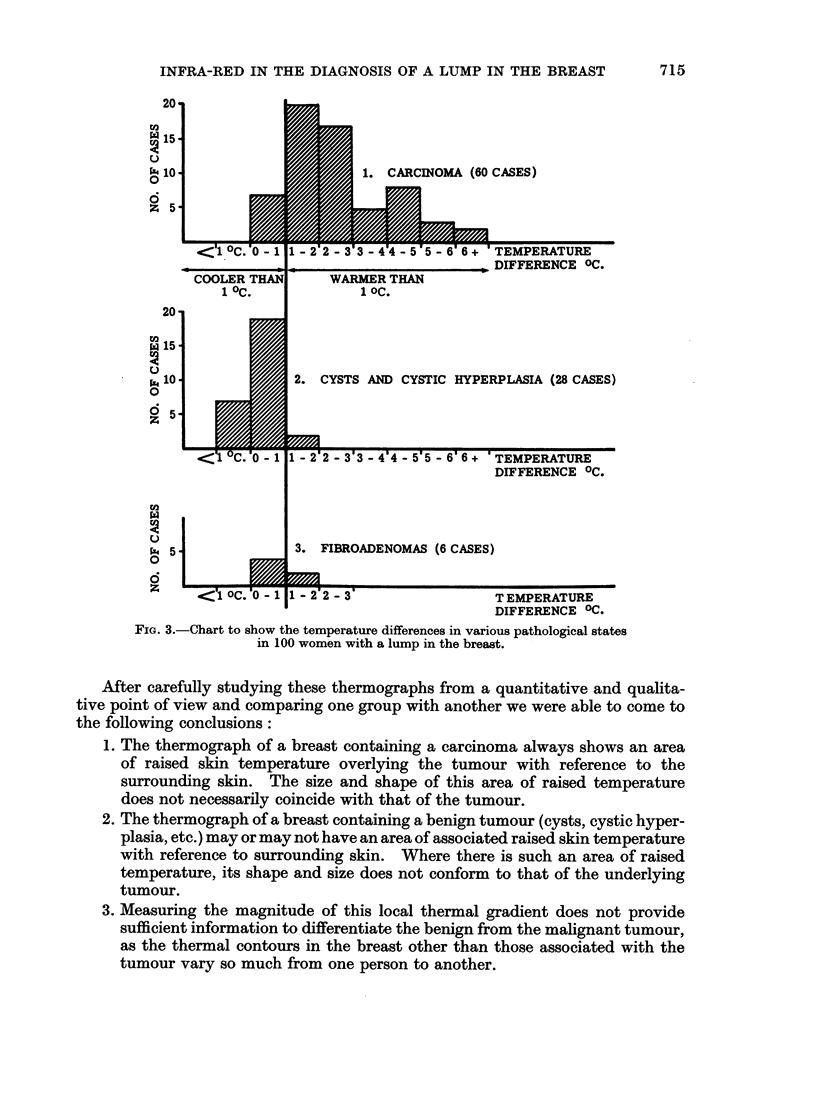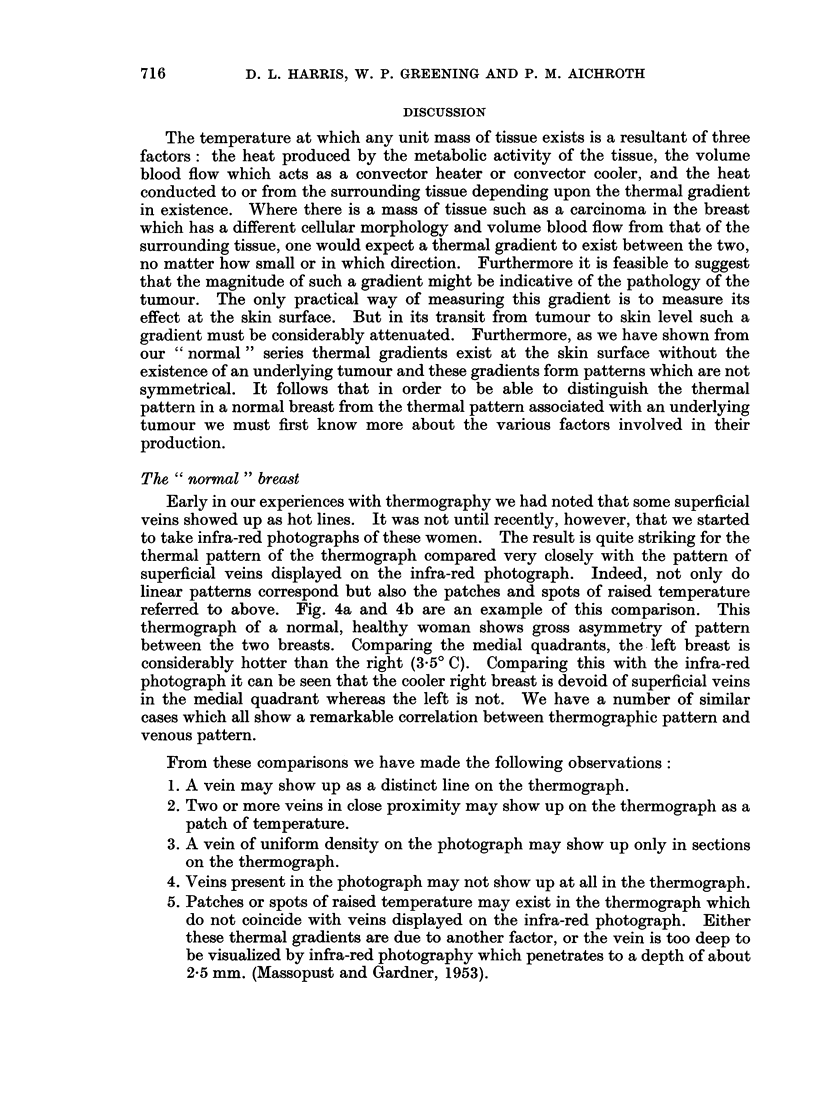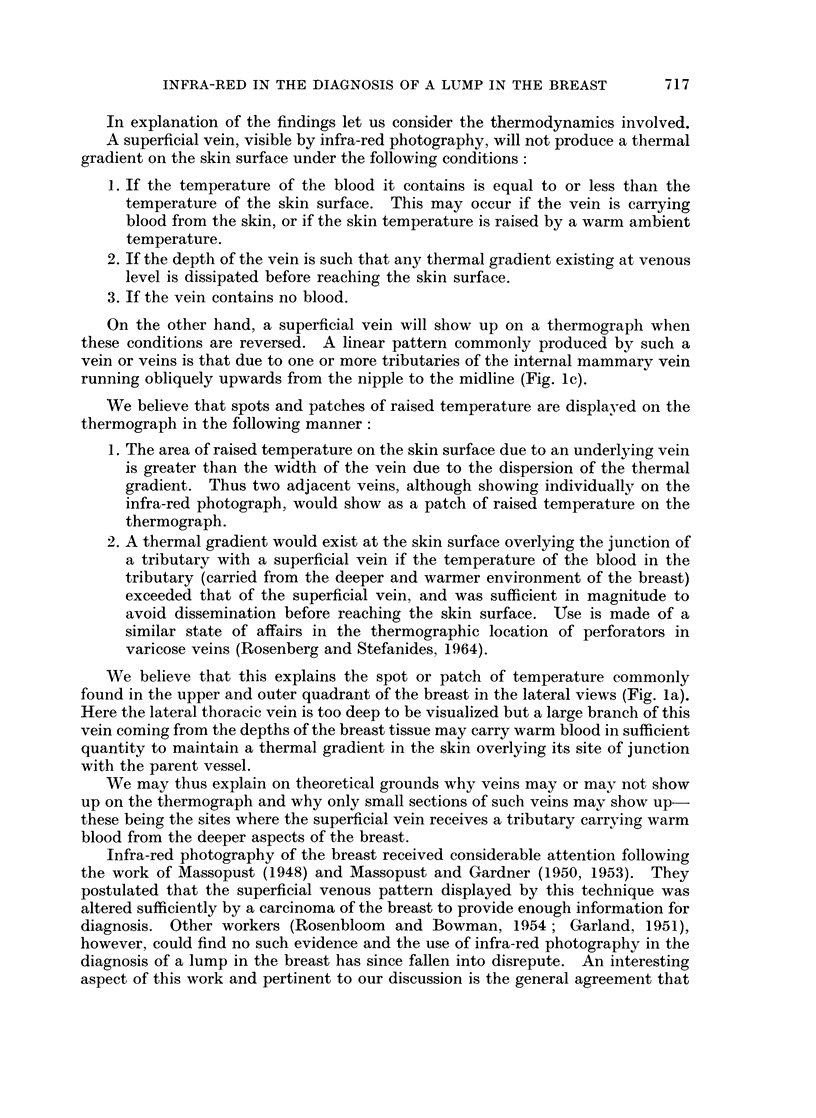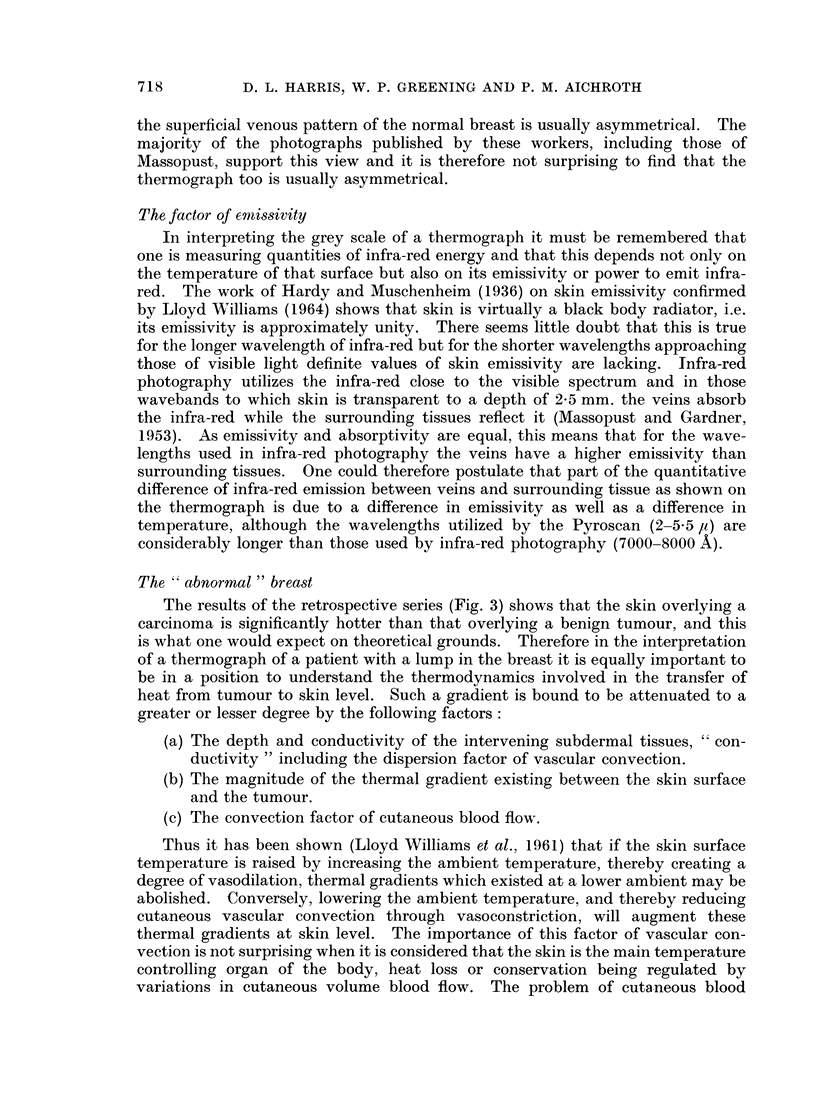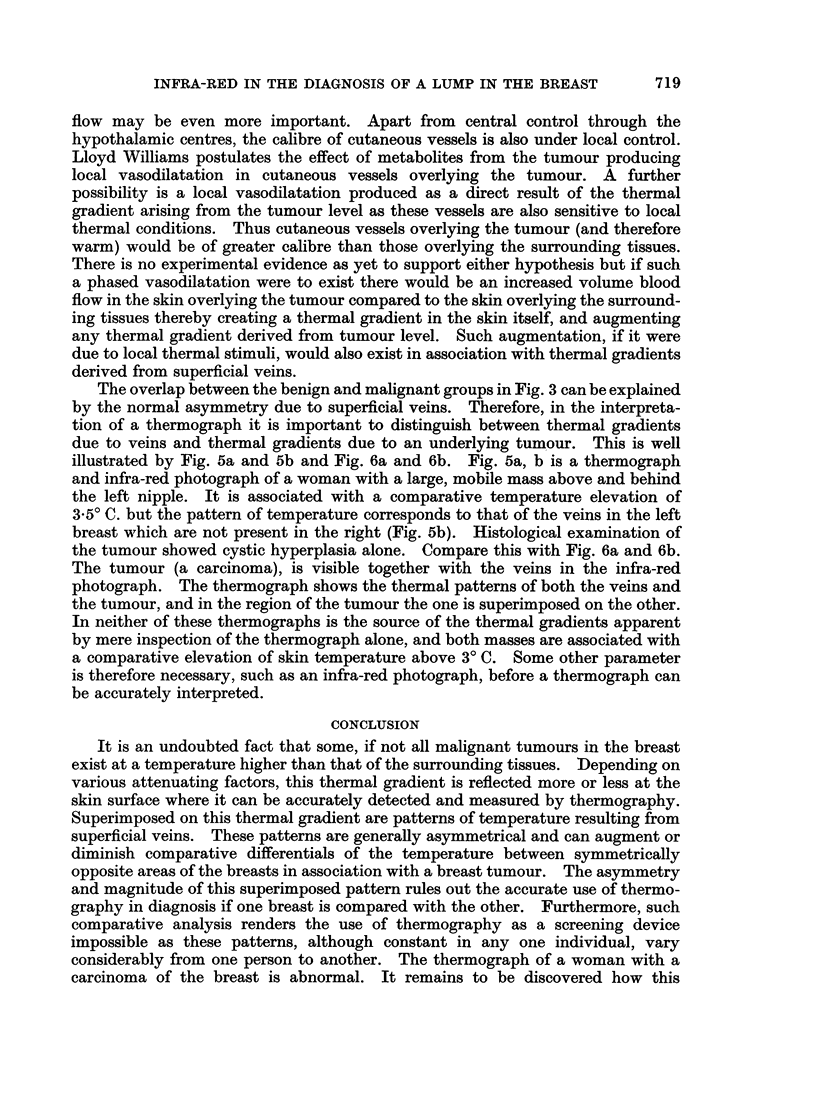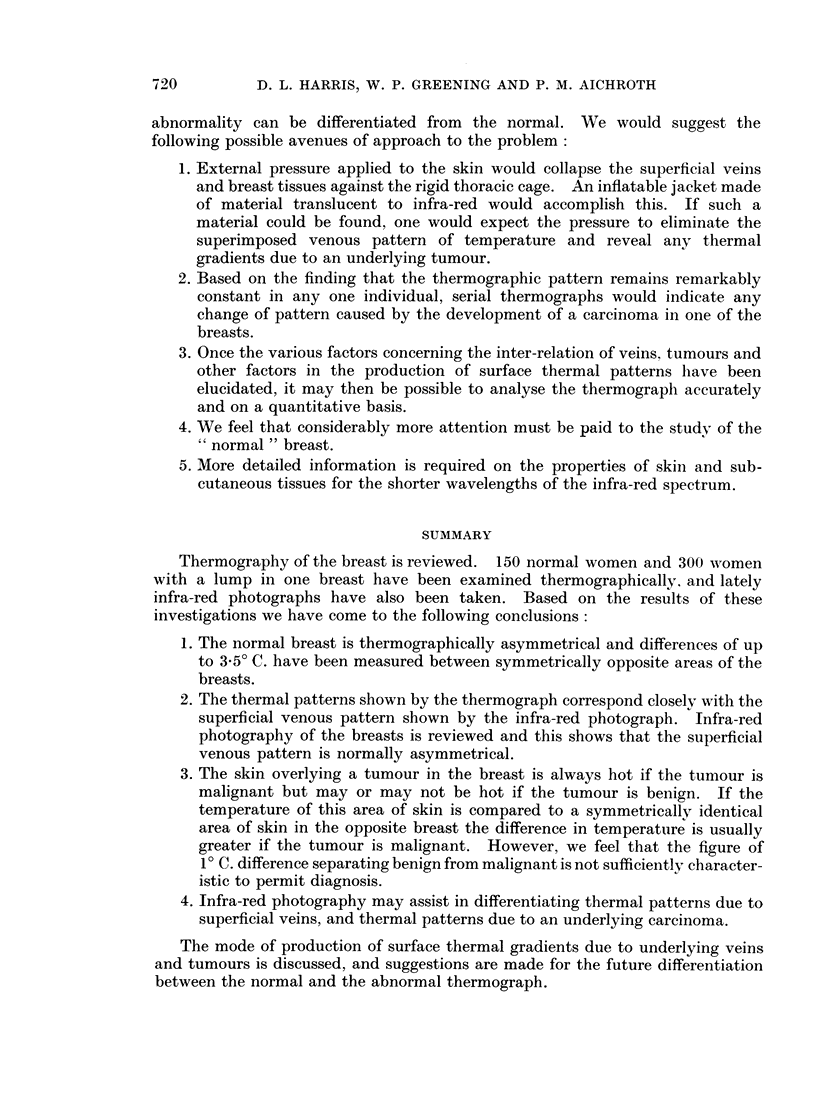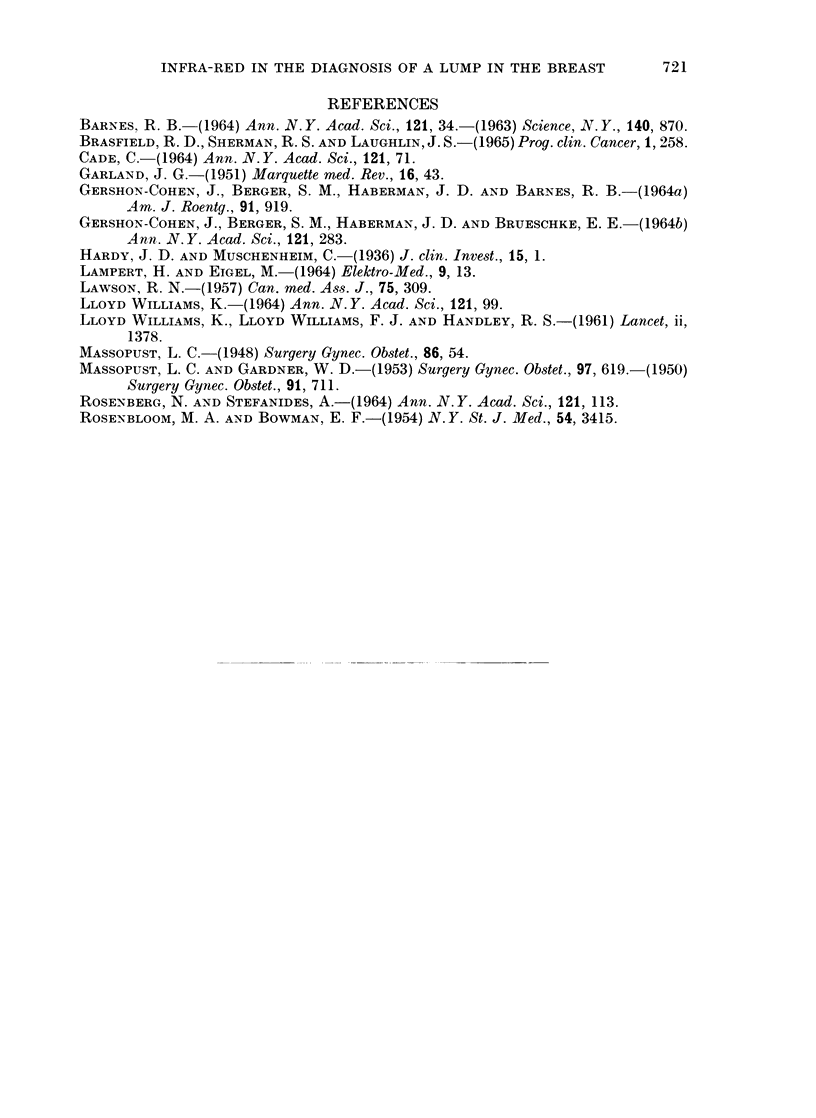# Infra-red in the diagnosis of a lump in the breast.

**DOI:** 10.1038/bjc.1966.82

**Published:** 1966-12

**Authors:** D. L. Harris, W. P. Greening, P. M. Aichroth

## Abstract

**Images:**


### INFRA-RED IN THE DIAGNOSIS OF A LUMP IN THE BREAST

D. L. HARRIS*, W. P. GREENING AND P. M. AICHROTH

*From the Breast Unit, Royal Marsden Hospital, London, S.W.3*

Received for publication October 17, 1966

ANY system of diagnosis depends upon the detection, measurement and assimilation of properties which are characteristic of the pathological process involved. These may be either physical, chemical or biological. Attention has been drawn, recently, to the finding that malignant tumours in the breast are hotter than benign tumours, and it has been suggested that this difference may be sufficiently characteristic to permit a pre-operative diagnosis. The accurate detection and mensuration of this temperature elevation is therefore fundamental if such a criterion is to be used in diagnosis.

As it is impractical, and virtually impossible, to obtain an accurate assessment of thermal conditions at tumour level, the thermal conditions at the skin surface overlying the tumour have been used with the contralateral uninvolved breast as a comparative control.

To measure the thermal gradients at the skin surface one may either use heat conducted to the instrument as with a thermocouple, or heat radiated to the instrument. The latter method depends upon the Stephan-Boltzman law which states that the quantity of infra-red emitted by a surface varies directly with the fourth power of its temperature in degrees Absolute and the emissivity of the surface. A full account of the physics involved in infra-red thermometry is given by Barnes (1964). Such infra-red thermometers may be either hand held whereby a small area of temperature is measured, or incorporated into an optical scanning system whereby the thermal contours of a large surface area are measured. Such measurements are usually recorded in the form of a grey scale, and if this is calibrated by including a series of black bodies of known temperature with the scan, the temperature of any spot on the recording or thermograph can be interpreted by measuring the greyness with a densitometer.

Lawson (1957) was the first to detect an elevated skin temperature overlying a malignant tumour in the breast when compared to a symmetrically identical area in the opposite breast. He did not find such an elevation if the tumour were benign.

Lloyd Williams *et al.* (1961) repeated Lawson's observations on a series of 100 women with a lump in one breast. They found that 54 out of 57 carcinomas were associated with a temperature elevation of over 1° C. and 23 out of 24 cysts and adenomas were associated with a thermal difference of less than 1° C. They used a hand held infra-red detector, the Schwarz thermopile and a comparative assessment of the thermal contours of the skin between right and left breasts required the charting of a series of individual measurements on a proforma. By using such

* Present address : Mulago Hospital, Kampala, Uganda.

a standard proforma inaccuracies are bound to occur when one considers the variable shape and size of the female breast.

When clinical infra-red scanning machines were introduced this difficulty was overcome for the thermograph was an exact and permanent record of the thermal contours existing at the time of recording and also an exact replica of the anatomical shape and size of the breasts. The method is also much quicker than plotting a temperature chart with a hand held instrument and thereby fluctuations in factors affecting the thermal contour of the skin surface other than that due to the underlying tumour are reduced (Barnes, 1963).

Several workers have reported their experiences using such an infra-red scanning device for the diagnosis of the lump in the breast—Gershon-Cohen *et al.* (1964*a*, *b*), Lloyd Williams *et al.* (1961), Brasfield *et al.* (1965). The criteria upon which these workers base their distinction between benign and malignant remains the same, namely a difference of 1° C. between the two breasts. Brasfield *et al.* (1965), in a series of 38 carcinomas, reports 25 as positive, 6 as negative and 7 as equivocal, and of 75 benign tumours, 50 were reported as normal, 10 were associated with an increase in temperature and 15 were equivocal. Little attention is paid to the accuracy of measurement in these cases and this can vary considerably depending upon the range of temperature over which the grey scale of the recording is extended (Gershon-Cohen *et al.*, 1964*b*).

A third method of infra-red thermometry is that described by Lampert and Eigel (1964). These workers have devised an apparatus where the patient stands within a blackened cylinder while a lead sulphide detector describes a spiral circuit around her. The pitch of the spiral is 1 cm. In this way a series of tracings is produced according to the thermal contours crossed by the path of the detector. Asymmetry of pattern is thus immediately apparent. They report a case of breast cancer associated with an asymmetrical rise in temperature, but they also found unexplained asymmetry of thermal contours in some normal individuals.

In all the reported series using either spot temperature measurements or thermographs it appears that a comparative elevation of 1° C. is not sufficiently specific to permit accurate differentiation between benign and malignant. It must also be remembered that most of these series are of necessity retrospective in that the results of thermometry are compared with the results of histological examination of the tumours. Such a retrospective study gives no indication of the value of the method for diagnostic purposes, but merely indicates criteria that may be of diagnostic value. Only by applying these criteria to a prospective series, where histology is predicted by thermometry, can a true assay of the potentials of the method be made.

In assessing the value of thermometry it appears that a fundamental factor has been overlooked. Very little attention has been paid to the thermal patterns existing in the breasts of a normal, healthy female, and it is impossible to draw conclusions of abnormality without first having a background knowledge of normality.

The application of infra-red thermometry to the diagnosis of the lump in the breast has been studied at this Hospital over the past three years. We report here our own observations on both normal and abnormal women and discuss the method of interpretation of the thermograph of the female breast in the light of these results.

### EXPERIMENTAL DATA

*Instruments.*—A Schwarz thermopile (Lloyd Williams *et al.*, 1961) is used for spot temperature measurements.

Thermographs are taken with the Smith's Pyroscan Mark IIb with a modified pre-amplifier and a 2 $\mu$ cut-on filter to exclude visible light. The Pyroscan (Cade, 1964) uses an indium antimonide photo-conductive cell cooled with liquid nitrogen and the amplified signal is recorded on Mufax Type H electro-sensitive paper. A standard black body temperature reference is included with each scan providing a calibrated grey scale. The reference is stable to fluctuations in ambient conditions. A densitometer is used to calculate the temperature from the grey scale of the thermograph. With the grey scale spread over 5° C. black to white it is estimated that the system is accurate to within 0·25° C. Most of the thermographs were taken at about this level of accuracy.

### *Ambient conditions*

All the patients are examined and scanned in an air conditioned room at a stable ambient temperature of 19° C. $\pm$ 0·5° C. A cooling time of 10 minutes is allowed for each patient before scanning (Gershon-Cohen *et al.*, 1964*a*, *b*), and while the thermograph is being taken the patient sits in a specially designed chair with the arms held on rests at right angles to the body. The chair is capable of rotation about the axis of the trunk so that identically comparable lateral and oblique views can be taken of the breasts. An anterior and two lateral views are normally taken of each patient. The scanning time for the three views is about 5 minutes in total. This speed of scan allows the amp gain and contrast controls to be adjusted for each patient so that maximum use of the grey scale is made.

### *Patients*

150 normal women volunteers have been seen. These have come mainly from local Family Planning Clinics and through our Cervical Smear Clinic.

Over 300 women with a lump in one breast have also been seen during the past three years. Of these, thermographs have been taken during the past year of 150. The earlier cases were examined with the Schwarz thermopile only.

All the women were required to answer a proforma detailing menstrual history, obstetric and lactation history, current medications including oral contraceptives and details of any past breast trouble. A thorough clinical examination of the breast was recorded in each case.

" *Normal* " *women.*—Note must be made of the term " normal ". This can only be judged on clinical grounds unless one is to subject all female volunteers to biopsy of areas of lumpiness. Most clinicians will agree that a uniformly soft breast is unusual. Most women have one or more areas of what is generally termed " fibro-adenosis ". Such lumpiness or granularity due to the phases of hyperplasia-involution must therefore be regarded as within normal limits in such a series.

### *Infra-red photography*

Recently, infra-red photographs have been taken of both " normal " and " abnormal " women. These were taken in the Photographic Department of the Hospital which is not under controlled ambient conditions.

### RESULTS

*The " normal " breast.*—The interpretation of a thermograph may be either qualitative when the thermal pattern is examined, or quantitative when thermal differences are calculated between right and left breasts.

### *Thermal pattern*

After repeated examination of the 150 thermographs of normal women we can find no system of classification. The following observations have been made :

1. Complete symmetry of patterns between the breasts is rare, both in the anterior view and in the lateral views.
2. Areas of raised temperature may be represented as linear patterns, " patches " of heat, " spots " of heat, or a mosaic pattern.
3. Such areas of raised temperature are commonly found in two sites in the breast :
   (a) A linear pattern running obliquely upwards from the nipple to the sternum (Fig. 1c).
   (b) A spot or patch of heat in the upper and outer quadrant shown on the lateral views (Fig. 1a and 1b).
4. Several volunteers have had repeated thermographs. In all of these the thermal pattern has remained remarkably constant.

### *Thermal differences*

As symmetry of thermal pattern is rare, it follows that in the majority of thermographs one or more areas can be found where thermal differences exist between symmetrically opposite parts of the breasts. Thermal differences of up to 3·5° C. have been measured between such areas and differences of up to 2·0° C. are common. In approximately 50% of the thermographs there was asymmetry of areolar temperature up to 2·0° C. Figure 2 gives some idea of the maximum thermal differences existing between symmetrically opposite parts of the breast in 50 consecutive thermographs.

We can find no statistical correlation between breast temperature and variations in blood hormone levels produced by oral contraception and the menstrual cycle.

### *The " abnormal " breasts*

Fig. 3 shows the thermal differences in 100 consecutive patients presenting with a lump in one breast when the temperature of the skin overlying the mass was compared with the temperature of an exactly symmetrical area of skin in the opposite breast. These measurements were all made with the Schwarz thermopile and the results compare closely with those of Lloyd Williams *et al.* (1961).

In the 150 thermographs of women presenting with a lump in one breast only the latter 100 have been used for study as the initial 50 were taken while we were becoming familar with the operation of the Pyroscan and the quality of these thermographs was not sufficiently good. It was intended to use these thermographs in a prospective study, but after analysing the results of the normal women we felt that the criterion of 1° C. comparative difference as obtained from the

FIG. 2.—Graph showing the maximum symmetrical temperature difference between right and left breast in 50 normal women.

retrospective study was insufficient for accurate diagnosis. We therefore divided the thermographs according to the histological result as follows :

Carcinoma.
Cysts, cystic hyperplasia.
Fibroadenoma.
Simple fibrosis.
Lipoma.
Fat necrosis.
Sarcoma.

---

EXPLANATION OF PLATES.

FIG. 1.—Thermograph of a normal woman (who is wearing a necklace) showing some characteristic thermal patterns.

(a) Right lateral view.
(b) Left lateral view.
(c) Antero-posterior view.

FIG. 4

(a) Thermograph of a normal woman.
(b) Infra-red photograph of the same patient.

FIG. 5

(a) Thermograph of a patient with a cystic mass above and behind the left nipple.
(b) Infra-red photograph of the same patient.

FIG. 6

(a) Thermograph of a patient with a carcinoma in the upper quadrants of the left breast.
(b) Infra-red photograph of the same patient.

1a

1b

1c

Harris, Greening and Aichroth.

4a

4b

5a

5b

6a

6b

Harris, Greening and Aichroth.

FIG. 3.—Chart to show the temperature differences in various pathological states in 100 women with a lump in the breast.

After carefully studying these thermographs from a quantitative and qualitative point of view and comparing one group with another we were able to come to the following conclusions :

1. The thermograph of a breast containing a carcinoma always shows an area of raised skin temperature overlying the tumour with reference to the surrounding skin. The size and shape of this area of raised temperature does not necessarily coincide with that of the tumour.
2. The thermograph of a breast containing a benign tumour (cysts, cystic hyperplasia, etc.) may or may not have an area of associated raised skin temperature with reference to surrounding skin. Where there is such an area of raised temperature, its shape and size does not conform to that of the underlying tumour.
3. Measuring the magnitude of this local thermal gradient does not provide sufficient information to differentiate the benign from the malignant tumour, as the thermal contours in the breast other than those associated with the tumour vary so much from one person to another.

### DISCUSSION

The temperature at which any unit mass of tissue exists is a resultant of three factors : the heat produced by the metabolic activity of the tissue, the volume blood flow which acts as a convector heater or convector cooler, and the heat conducted to or from the surrounding tissue depending upon the thermal gradient in existence. Where there is a mass of tissue such as a carcinoma in the breast which has a different cellular morphology and volume blood flow from that of the surrounding tissue, one would expect a thermal gradient to exist between the two, no matter how small or in which direction. Furthermore it is feasible to suggest that the magnitude of such a gradient might be indicative of the pathology of the tumour. The only practical way of measuring this gradient is to measure its effect at the skin surface. But in its transit from tumour to skin level such a gradient must be considerably attenuated. Furthermore, as we have shown from our " normal " series thermal gradients exist at the skin surface without the existence of an underlying tumour and these gradients form patterns which are not symmetrical. It follows that in order to be able to distinguish the thermal pattern in a normal breast from the thermal pattern associated with an underlying tumour we must first know more about the various factors involved in their production.

### *The " normal " breast*

Early in our experiences with thermography we had noted that some superficial veins showed up as hot lines. It was not until recently, however, that we started to take infra-red photographs of these women. The result is quite striking for the thermal pattern of the thermograph compared very closely with the pattern of superficial veins displayed on the infra-red photograph. Indeed, not only do linear patterns correspond but also the patches and spots of raised temperature referred to above. Fig. 4a and 4b are an example of this comparison. This thermograph of a normal, healthy woman shows gross asymmetry of pattern between the two breasts. Comparing the medial quadrants, the left breast is considerably hotter than the right (3·5° C). Comparing this with the infra-red photograph it can be seen that the cooler right breast is devoid of superficial veins in the medial quadrant whereas the left is not. We have a number of similar cases which all show a remarkable correlation between thermographic pattern and venous pattern.

From these comparisons we have made the following observations :

1. A vein may show up as a distinct line on the thermograph.
2. Two or more veins in close proximity may show up on the thermograph as a patch of temperature.
3. A vein of uniform density on the photograph may show up only in sections on the thermograph.
4. Veins present in the photograph may not show up at all in the thermograph.
5. Patches or spots of raised temperature may exist in the thermograph which do not coincide with veins displayed on the infra-red photograph. Either these thermal gradients are due to another factor, or the vein is too deep to be visualized by infra-red photography which penetrates to a depth of about 2·5 mm. (Massopust and Gardner, 1953).

In explanation of the findings let us consider the thermodynamics involved. A superficial vein, visible by infra-red photography, will not produce a thermal gradient on the skin surface under the following conditions :

1. If the temperature of the blood it contains is equal to or less than the temperature of the skin surface. This may occur if the vein is carrying blood from the skin, or if the skin temperature is raised by a warm ambient temperature.
2. If the depth of the vein is such that any thermal gradient existing at venous level is dissipated before reaching the skin surface.
3. If the vein contains no blood.

On the other hand, a superficial vein will show up on a thermograph when these conditions are reversed. A linear pattern commonly produced by such a vein or veins is that due to one or more tributaries of the internal mammary vein running obliquely upwards from the nipple to the midline (Fig. 1c).

We believe that spots and patches of raised temperature are displayed on the thermograph in the following manner :

1. The area of raised temperature on the skin surface due to an underlying vein is greater than the width of the vein due to the dispersion of the thermal gradient. Thus two adjacent veins, although showing individually on the infra-red photograph, would show as a patch of raised temperature on the thermograph.
2. A thermal gradient would exist at the skin surface overlying the junction of a tributary with a superficial vein if the temperature of the blood in the tributary (carried from the deeper and warmer environment of the breast) exceeded that of the superficial vein, and was sufficient in magnitude to avoid dissemination before reaching the skin surface. Use is made of a similar state of affairs in the thermographic location of perforators in varicose veins (Rosenberg and Stefanides, 1964).

We believe that this explains the spot or patch of temperature commonly found in the upper and outer quadrant of the breast in the lateral views (Fig. 1a). Here the lateral thoracic vein is too deep to be visualized but a large branch of this vein coming from the depths of the breast tissue may carry warm blood in sufficient quantity to maintain a thermal gradient in the skin overlying its site of junction with the parent vessel.

We may thus explain on theoretical grounds why veins may or may not show up on the thermograph and why only small sections of such veins may show up—these being the sites where the superficial vein receives a tributary carrying warm blood from the deeper aspects of the breast.

Infra-red photography of the breast received considerable attention following the work of Massopust (1948) and Massopust and Gardner (1950, 1953). They postulated that the superficial venous pattern displayed by this technique was altered sufficiently by a carcinoma of the breast to provide enough information for diagnosis. Other workers (Rosenbloom and Bowman, 1954 ; Garland, 1951), however, could find no such evidence and the use of infra-red photography in the diagnosis of a lump in the breast has since fallen into disrepute. An interesting aspect of this work and pertinent to our discussion is the general agreement that the superficial venous pattern of the normal breast is usually asymmetrical. The majority of the photographs published by these workers, including those of Massopust, support this view and it is therefore not surprising to find that the thermograph too is usually asymmetrical.

*The factor of emissivity*

In interpreting the grey scale of a thermograph it must be remembered that one is measuring quantities of infra-red energy and that this depends not only on the temperature of that surface but also on its emissivity or power to emit infra-red. The work of Hardy and Muschenheim (1936) on skin emissivity confirmed by Lloyd Williams (1964) shows that skin is virtually a black body radiator, i.e. its emissivity is approximately unity. There seems little doubt that this is true for the longer wavelength of infra-red but for the shorter wavelengths approaching those of visible light definite values of skin emissivity are lacking. Infra-red photography utilizes the infra-red close to the visible spectrum and in those wavebands to which skin is transparent to a depth of 2·5 mm. the veins absorb the infra-red while the surrounding tissues reflect it (Massopust and Gardner, 1953). As emissivity and absorptivity are equal, this means that for the wavelengths used in infra-red photography the veins have a higher emissivity than surrounding tissues. One could therefore postulate that part of the quantitative difference of infra-red emission between veins and surrounding tissue as shown on the thermograph is due to a difference in emissivity as well as a difference in temperature, although the wavelengths utilized by the Pyroscan (2–5·5 $\mu$) are considerably longer than those used by infra-red photography (7000–8000 Å).

*The " abnormal " breast*

The results of the retrospective series (Fig. 3) shows that the skin overlying a carcinoma is significantly hotter than that overlying a benign tumour, and this is what one would expect on theoretical grounds. Therefore in the interpretation of a thermograph of a patient with a lump in the breast it is equally important to be in a position to understand the thermodynamics involved in the transfer of heat from tumour to skin level. Such a gradient is bound to be attenuated to a greater or lesser degree by the following factors :

(a) The depth and conductivity of the intervening subdermal tissues, " conductivity " including the dispersion factor of vascular convection.
(b) The magnitude of the thermal gradient existing between the skin surface and the tumour.
(c) The convection factor of cutaneous blood flow.

Thus it has been shown (Lloyd Williams *et al.*, 1961) that if the skin surface temperature is raised by increasing the ambient temperature, thereby creating a degree of vasodilation, thermal gradients which existed at a lower ambient may be abolished. Conversely, lowering the ambient temperature, and thereby reducing cutaneous vascular convection through vasoconstriction, will augment these thermal gradients at skin level. The importance of this factor of vascular convection is not surprising when it is considered that the skin is the main temperature controlling organ of the body, heat loss or conservation being regulated by variations in cutaneous volume blood flow. The problem of cutaneous blood flow may be even more important. Apart from central control through the hypothalamic centres, the calibre of cutaneous vessels is also under local control. Lloyd Williams postulates the effect of metabolites from the tumour producing local vasodilatation in cutaneous vessels overlying the tumour. A further possibility is a local vasodilatation produced as a direct result of the thermal gradient arising from the tumour level as these vessels are also sensitive to local thermal conditions. Thus cutaneous vessels overlying the tumour (and therefore warm) would be of greater calibre than those overlying the surrounding tissues. There is no experimental evidence as yet to support either hypothesis but if such a phased vasodilatation were to exist there would be an increased volume blood flow in the skin overlying the tumour compared to the skin overlying the surrounding tissues thereby creating a thermal gradient in the skin itself, and augmenting any thermal gradient derived from tumour level. Such augmentation, if it were due to local thermal stimuli, would also exist in association with thermal gradients derived from superficial veins.

The overlap between the benign and malignant groups in Fig. 3 can be explained by the normal asymmetry due to superficial veins. Therefore, in the interpretation of a thermograph it is important to distinguish between thermal gradients due to veins and thermal gradients due to an underlying tumour. This is well illustrated by Fig. 5a and 5b and Fig. 6a and 6b. Fig. 5a, b is a thermograph and infra-red photograph of a woman with a large, mobile mass above and behind the left nipple. It is associated with a comparative temperature elevation of 3·5° C. but the pattern of temperature corresponds to that of the veins in the left breast which are not present in the right (Fig. 5b). Histological examination of the tumour showed cystic hyperplasia alone. Compare this with Fig. 6a and 6b. The tumour (a carcinoma), is visible together with the veins in the infra-red photograph. The thermograph shows the thermal patterns of both the veins and the tumour, and in the region of the tumour the one is superimposed on the other. In neither of these thermographs is the source of the thermal gradients apparent by mere inspection of the thermograph alone, and both masses are associated with a comparative elevation of skin temperature above 3° C. Some other parameter is therefore necessary, such as an infra-red photograph, before a thermograph can be accurately interpreted.

### CONCLUSION

It is an undoubted fact that some, if not all malignant tumours in the breast exist at a temperature higher than that of the surrounding tissues. Depending on various attenuating factors, this thermal gradient is reflected more or less at the skin surface where it can be accurately detected and measured by thermography. Superimposed on this thermal gradient are patterns of temperature resulting from superficial veins. These patterns are generally asymmetrical and can augment or diminish comparative differentials of the temperature between symmetrically opposite areas of the breasts in association with a breast tumour. The asymmetry and magnitude of this superimposed pattern rules out the accurate use of thermography in diagnosis if one breast is compared with the other. Furthermore, such comparative analysis renders the use of thermography as a screening device impossible as these patterns, although constant in any one individual, vary considerably from one person to another. The thermograph of a woman with a carcinoma of the breast is abnormal. It remains to be discovered how this abnormality can be differentiated from the normal. We would suggest the following possible avenues of approach to the problem :

1. External pressure applied to the skin would collapse the superficial veins and breast tissues against the rigid thoracic cage. An inflatable jacket made of material translucent to infra-red would accomplish this. If such a material could be found, one would expect the pressure to eliminate the superimposed venous pattern of temperature and reveal any thermal gradients due to an underlying tumour.
2. Based on the finding that the thermographic pattern remains remarkably constant in any one individual, serial thermographs would indicate any change of pattern caused by the development of a carcinoma in one of the breasts.
3. Once the various factors concerning the inter-relation of veins, tumours and other factors in the production of surface thermal patterns have been elucidated, it may then be possible to analyse the thermograph accurately and on a quantitative basis.
4. We feel that considerably more attention must be paid to the study of the "normal" breast.
5. More detailed information is required on the properties of skin and subcutaneous tissues for the shorter wavelengths of the infra-red spectrum.

### SUMMARY

Thermography of the breast is reviewed. 150 normal women and 300 women with a lump in one breast have been examined thermographically, and lately infra-red photographs have also been taken. Based on the results of these investigations we have come to the following conclusions :

1. The normal breast is thermographically asymmetrical and differences of up to 3·5° C. have been measured between symmetrically opposite areas of the breasts.
2. The thermal patterns shown by the thermograph correspond closely with the superficial venous pattern shown by the infra-red photograph. Infra-red photography of the breasts is reviewed and this shows that the superficial venous pattern is normally asymmetrical.
3. The skin overlying a tumour in the breast is always hot if the tumour is malignant but may or may not be hot if the tumour is benign. If the temperature of this area of skin is compared to a symmetrically identical area of skin in the opposite breast the difference in temperature is usually greater if the tumour is malignant. However, we feel that the figure of 1° C. difference separating benign from malignant is not sufficiently characteristic to permit diagnosis.
4. Infra-red photography may assist in differentiating thermal patterns due to superficial veins, and thermal patterns due to an underlying carcinoma.

The mode of production of surface thermal gradients due to underlying veins and tumours is discussed, and suggestions are made for the future differentiation between the normal and the abnormal thermograph.

### REFERENCES

BARNES, R. B.—(1964) *Ann. N.Y. Acad. Sci.*, **121**, 34.—(1963) *Science, N.Y.*, **140**, 870.

BRASFIELD, R. D., SHERMAN, R. S. AND LAUGHLIN, J. S.—(1965) *Prog. clin. Cancer*, **1**, 258.

CADE, C.—(1964) *Ann. N.Y. Acad. Sci.*, **121**, 71.

GARLAND, J. G.—(1951) *Marquette med. Rev.*, **16**, 43.

GERSHON-COHEN, J., BERGER, S. M., HABERMAN, J. D. AND BARNES, R. B.—(1964*a*) *Am. J. Roentg.*, **91**, 919.

GERSHON-COHEN, J., BERGER, S. M., HABERMAN, J. D. AND BRUESCHKE, E. E.—(1964*b*) *Ann. N.Y. Acad. Sci.*, **121**, 283.

HARDY, J. D. AND MUSCHENHEIM, C.—(1936) *J. clin. Invest.*, **15**, 1.

LAMPERT, H. AND EIGEL, M.—(1964) *Elektro-Med.*, **9**, 13.

LAWSON, R. N.—(1957) *Can. med. Ass. J.*, **75**, 309.

LLOYD WILLIAMS, K.—(1964) *Ann. N.Y. Acad. Sci.*, **121**, 99.

LLOYD WILLIAMS, K., LLOYD WILLIAMS, F. J. AND HANDLEY, R. S.—(1961) *Lancet*, ii, 1378.

MASSOPUST, L. C.—(1948) *Surgery Gynec. Obstet.*, **86**, 54.

MASSOPUST, L. C. AND GARDNER, W. D.—(1953) *Surgery Gynec. Obstet.*, **97**, 619.—(1950) *Surgery Gynec. Obstet.*, **91**, 711.

ROSENBERG, N. AND STEFANIDES, A.—(1964) *Ann. N.Y. Acad. Sci.*, **121**, 113.

ROSENBLOOM, M. A. AND BOWMAN, E. F.—(1954) *N.Y. St. J. Med.*, **54**, 3415.